# Comparison of the ability of HOMA-IR, VAI, and TyG indexes to predict metabolic syndrome in children with obesity: a cross-sectional study

**DOI:** 10.1186/s12887-023-03892-8

**Published:** 2023-02-11

**Authors:** Cihad Dundar, Ozlem Terzi, Hatice Nilden Arslan

**Affiliations:** Dept. of Public Health, Faculty of Medicine, Ondokuz Mayıs University, Samsun, Turkey

**Keywords:** Children, HOMA-IR, Metabolic syndrome, Obesity, Triglyceride-glucose index, Visceral adiposity index

## Abstract

**Background:**

The increasing trend in childhood obesity needs to be closely monitored and intervened due to long-term health issues such as metabolic syndrome, cardiovascular diseases, hypertension, and type 2 diabetes. We aimed to determine and compare the cut-off values for the visceral adiposity index (VAI), triglyceride-glucose index (TyG), and HOMA-IR for predicting metabolic syndrome (MetS).

**Methods:**

This population-based cross-sectional study was conducted in May 2019 in Samsun, Turkey. The study included 169 children with obesity aged 9 and 10 years. After anthropometric and clinical evaluation, fasting blood samples were collected from the children. The areas under the curve of the visceral adiposity index, triglyceride-glucose index, and HOMA-IR were compared by receiver-operating-characteristic (ROC) analysis in predicting the MetS.

**Results:**

The total prevalence of MetS was 21.3% in children with obesity, and it was two times higher in girls than in boys. The mean values of TyG and VAI were significantly different in those who have and have not MetS in both genders. However, the HOMA-IR index was higher only in girls with MetS and did not differ in boys by having MetS. The VAI and the TyG index both had statistically significant cut-off values in both sexes and a larger ROC area than the HOMA-IR index in predicting MetS.

**Conclusions:**

The VAI and TyG index are effective indicators in assessing the MetS risk in children with obesity. Both indexes can be considered useful tools in pediatric research and the evaluation of interventions. However, the HOMA-IR index formula needs to be developed taking into account age, gender, and ethnicity.

## Introduction

Obesity is a global health problem for children and adolescents due to the many morbid consequences it is associated with, and the increasing trend in childhood obesity needs to be closely monitored and intervened [1, 2]. Childhood obesity causes many short and long-term health issues such as metabolic syndrome (MetS), cardiovascular diseases, hypertension, type 2 diabetes, fatty liver, orthopedic difficulties, and decreased self-confidence [3, 4]. While MetS increases Type 2 diabetes risk through dysregulated cellular metabolism leading to insulin resistance, it also contributes to cardiovascular disease risk due to increased blood pressure, free fatty acids, triglycerides, and decreased HDL cholesterol levels. For these reasons, MetS is considered an indicator of future type 2 diabetes in children [5]. MetS, whose prevalence is increased by obesity, can persist into adulthood, and follow-up studies have shown that 50–80% of children with obesity become obese adults with a high risk of developing the aforementioned morbidities [6]. While the exact prevalence of pediatric MetS is unknown because of the wide range of physical and hormonal changes according to age and sex during childhood and adolescence [7], pediatric MetS is considered an important target for potential interventions to reduce the burden of cardiometabolic diseases [5]. Detecting MetS in children can provide an opportunity for early intervention to prevent CVD in adults [6]. However, calculating the risk of MetS requires various measurements such as waist circumference, triglyceride, HDL cholesterol, fasting glucose, and systolic and diastolic blood pressure levels [8]. Besides, lack of consensus on the limit value of the biochemical parameters used in calculating the risk of MetS in children, and missing national and/or international assessment systems for diagnosing MetS in children makes it difficult to use these indices [9].

As in adults, early detection of insulin resistance (IR) in children is important to prevent the development of cardiometabolic diseases such as Type 2 Diabetes Mellitus, metabolic syndrome, and coronary heart disease. The Homeostatic Model Assessment for Insulin Resistance (HOMA-IR) index, which has high sensitivity and specificity, has been shown to be more useful in estimating the risk of insulin resistance, instead of more invasive, complex, and expensive direct tests such as pancreatic suppression test and hyperinsulinemic-euglycemic glucose clamp technique[10]. HOMA-IR is an approved method for evaluating insulin resistance using fasting glucose and insulin levels and is widely used in clinical practice [11].

Visceral adiposity index (VAI) which is used to calculate cardiometabolic risk by anthropometric measurements and blood lipid parameters is a useful tool for detecting MetS in children and adolescents [12–15]. Recently, the use of the triglyceride glucose (TyG) index, a product of fasting glucose and triglyceride and a useful predictor of cardiometabolic risk factors in childhood and adolescence has also increased [16–19], because, they are more easily accessible assessment tools both at the outpatient clinics and at the community level, and are low cost [20]. Although VAI and TyG have proven to be useful in determining the risk of cardiometabolic disease in the adult population, few studies have been conducted on children with obesity. Therefore, we aimed to establish the cut-off points of the VAI, the TyG index, and the HOMA-IR index for the diagnosis of MetS in obese Turkish children, and to determine which of these indices will provide a more accurate approach to diagnosis.

## Methods

### Study design

To determine the prevalence of childhood obesity, we conducted a cross-sectional study on 9786 children representing all public and private primary school students between October and November 2016, in Samsun, Turkey [21]. This representative sample was selected from 70,660 students in 388 primary schools by multistage and stratified random sampling method by residency (rural/urban), school type (public/private), grade (I-IV), and gender. Classes with less than ten students were not included in the sample selection. The prevalence of obesity was found 10.5% (1030 children). While 403 of these children were first and second-grade students, 627 were third and fourth-grade students. We planned the current cross-sectional study to gather new data on the development of metabolic syndrome in children with obesity. However, the preparation of the project, sourcing and obtaining the approval of the ethics committee took two years, and those 627 students in the third and fourth grades had already graduated by then. In our country, especially in rural, students usually change their schools and leave their provinces or districts when they start secondary school, which makes it extremely hard to reach them again. For this reason, the students to be followed were limited to the 403 first and second-grade students who participated in the prior study. In addition, the lack of consensus on the definition of MetS in children under 9 years of age was another limiting factor in the selection of the study group [20].

### Sampling

We tried to reach the parents of 403 children by phone or e-mail based on information extracted from the records of the initial study in April and May 2019. Nineteen children had been lost during the follow-up period. The remaining students were invited to the family health center for re-examination. Children who were diagnosed with obesity in the previous study and whose BMI value is above two standard deviations according to the WHO's age and sex-specific BMI table were included in the study. Children who lost weight and were no longer obese (*n* = 14), and those who were medicating for obesity or for another metabolic disease (such as neurologic, respiratory, or cardiovascular disease) (*n* = 15) were excluded. We used the formula *n* = Nxt^2^xpxq/d^2^x(N − 1) + t^2^xpxq to determine the minimum sample size [22]. In the formula;

n = The minimum number of individuals to be sampled,

N = The number of individuals in the target group from which the sample will be taken (355),

p = Prevalence of the investigated event (MetS prevalence 30% in Turkish obese children),

q = 1-prevalence (70%),

t = The theoretical value found according to the “t” table at 5% significance level (1.96),

d = The accepted sampling error (0.05).

The minimum sample size was found *n* = (355)x(1.96)^2^x(0.30 × 0.70)/(0.05)^2^x(355–1) + (1.96)^2^x(0.3 × 0.7) = 169.

Since taking a blood sample is an invasive method and is frightening for the pediatric age group, we preferred to limit the number of participants to a minimum sample size. The current study group was selected from the list of children with obesity by a systematic sampling method.

### Anthropometrics and clinical assessment

For anthropometric measurements, children were dressed in light underwear and bare feet or in socks. The waist circumference (WC) measurements were taken at the umbilical level. The student's height and weight were measured using a stadiometer with a sensitivity of ± 0.1 cm (SECA Ltd, Medical Scales and Measurement Systems, Birmingham, United Kingdom) and a calibrated weighing instrument with a sensitivity of ± 0·1 kg (Seca Mess und Wiegetechnik, Germany), respectively. The height and weight were measured twice, and the averages were recorded. The BMI was calculated by the Quetelet equation (weight / height^2^). According to the WHO BMI chart, the children who had a BMI score above two standard deviations for age and sex were defined as obese [23]. Systolic (SBP) and diastolic blood pressures (DBP) were measured twice at five-minute intervals with the ERKA sphygmomanometer, and their average was recorded as mm Hg. Being a blood pressure higher than 130/85 mm Hg was accepted as hypertension [24].

### Biochemical analyses and definitions

The fasting blood samples of the children were collected between 08:00 and 09:00 h. The levels of total cholesterol (TC), low-density lipoprotein (LDL), high-density lipoprotein (HDL), triglyceride (TG), and fasting blood glucose (FBG) were measured. Biochemical measurements of fasting blood samples were studied with the COBAS 8000 c-702 (Roche) auto analyzer device using colorimetric kits.

Insulin resistance was calculated according to the Homeostasis Model Assessment insulin resistance (HOMA-IR) index. HOMA-IR was obtained with the formula fasting glucose (mmol/L) X fasting insulin (IU/ml) / 405. HOMA-IR values ​​above 3.54 were identified as insulin resistance [10]. The TyG index was calculated using the following formula: ln [fasting triglycerides (mg/dl) × fasting plasma glucose (mg/dL)/2] [25]. The VAI were calculated both for men and women separately according to the following Equations [13]:

$$\mathrm{Male }= [\mathrm{WC }/ (39.68 + (1.88\mathrm{ X BMI}))]\mathrm{ X }(\mathrm{TG }/ 1.03)\mathrm{ X }(1.31 /\mathrm{ HDL});$$

$$\mathrm{Female }= [\mathrm{WC }/ (36.58 + (1.89\mathrm{ X BMI}))]\mathrm{ X }(\mathrm{TG }/ 0.81)\mathrm{ X }(1.52 /\mathrm{ HDL})$$

According to the International Diabetes Federation’s criteria, the presence of at least two of the following criteria accompanying central obesity was considered MetS: Triglyceride level of ≥ 150 mg/dL, HDL cholesterol level of < 40 mg/dL, fasting glucose level equal to or higher than 100 mg/dL, systolic blood pressure of higher than 130 mm Hg, or diastolic blood pressure of equal to or higher than 85 mm Hg, or receiving antihypertensive treatment [9].

### Statistical analysis

Statistical analysis was performed by SPSS version 22·0 (IBM Corporation, Armonk, NY, USA) package program. The conformity of continuous variables to normal distribution was determined by the Shapiro Wilk test. Quantitative data were presented with arithmetic mean and standard deviation, and qualitative data were presented in numbers and percentages. The Chi-square test was used to compare categorical variables. While non-normally distributed data were compared using the Mann Whitney U test, the Student’s t-test was used to compare normally distributed quantitative data. The sensitivity, specificity, and cut-off points of the VAI and the TyG were examined using receiver-operating characteristic (ROC) curve analyses. We assessed the overall performance of each index for predicting the MetS by computing the area under the curve (AUC). We determined the best cut-off points as the point on the curve where both sensitivity and specificity were the highest. In statistical analysis, p˂0.05 was accepted as the limit value of significance.

## Results

The mean age of the 169 students with obesity was 10·2 ± 0·5 years, and 85 (50·3%) of them were girls. While the anthropometric parameters were no different except waist circumference according to gender, biochemical parameters were higher in girls than in boys, except HDL (Table 1). The total prevalence of MetS was 21.3% in children with obesity, and it was two times higher in girls than in boys. The TyG index was similar in both genders (*p* = 0.376).

**Table 1:** Anthropometrical and biochemical characteristics of the children with obesity (Mean ± SD)

Characteristics	Boys (*n* = 84)	Girls(*n* = 85)	*p*
Weight (kg)	56.4 ± 7.9	56.3 ± 7.7	0.222
Height (cm)	143.1 ± 6.5	144.6 ± 6.7	0.463
BMI (kg/m^2^)	27.4 ± 2.3	26.8 ± 2.4	0.062
WC (cm)	84.3 ± 8.2	81.1 ± 7.6	**0.007**
FBG (mmol/L)	5.1 ± 0.4	5.2 ± 0.5	0.415
Insulin (IU/ml)	18.1 ± 5.1	21.4 ± 7.5	**0.004**
Total cholesterol (mg/dl)	151.2 ± 29.4	146.1 ± 30.3	0.266
Triglyceride (mg/dl)	105.4 ± 57.8	109.4 ± 53.1	0.334
HDL (mg/dl)	48.7 ± 10.9	45.0 ± 8.6	**0.030**
LDL (mg/dl)	81.4 ± 25.7	79.1 ± 26.2	0.558
SBP (mmHg)	117.7 ± 9.8	118.8 ± 10.7	0.394
DBP (mmHg)	74.1 ± 8.7	74.3 ± 8.9	0.999
HOMA-IR	4.1 ± 1.2	5.0 ± 2.1	**0.007**
TyG	8.4 ± 0.5	8.4 ± 0.5	0.376
VAI	2.8 ± 2.1	4.4 ± 2.7	** < 0.001**
MetS	12 (14.3%)	24 (28.2%)	**0.031**

*BMI* Body Mass Index, *WC* Waist Circumference, *FBG* Fasting Blood Glucose, *HDL* High-density Lipoprotein, *LDL* Low-density Lipoprotein, *SBP* Systolic Blood Pressure, *DBP* Diastolic Blood Pressure, *HOMA-IR* Homeostasis Model Assessment Insulin Resistance, *TyG* Triglyceride-glucose index, *VAI* Visceral adiposity index, *MetS* Metabolic syndrome

HOMA-IR, TyG, and VAI were higher both in boys and girls with MetS than in those without (Table 2). However, HOMA-IR was statistically different in girls only.

**Table 2:** The HOMA-IR, the TyG, and the VAI values in boys and girls by having MetS criteria (Mean ± SD)

Indexes	Boys			Girls		
	**MetS ( +)**	**MetS (-)**	**p**	**MetS ( +)**	**MetS (-)**	***p***
HOMA-IR	4.36 ± 1.1	4.09 ± 1.2	0.245	6.15 ± 2.4	4.55 ± 1.8	0.001
TyG	8.77 ± 0.5	8.29 ± 0.5	0.010	8.79 ± 0.5	8.29 ± 0.4	< 0.001
VAI	5.11 ± 3.7	2.43 ± 1.5	0.002	6.88 ± 3.8	3.64 ± 1.7	< 0.001

*MetS* Metabolic syndrome, *HOMA-IR* Homeostasis Model Assessment Insulin Resistance, *TyG* Triglyceride-glucose index, *VAI* Visceral adiposity index

**Fig. 1:**
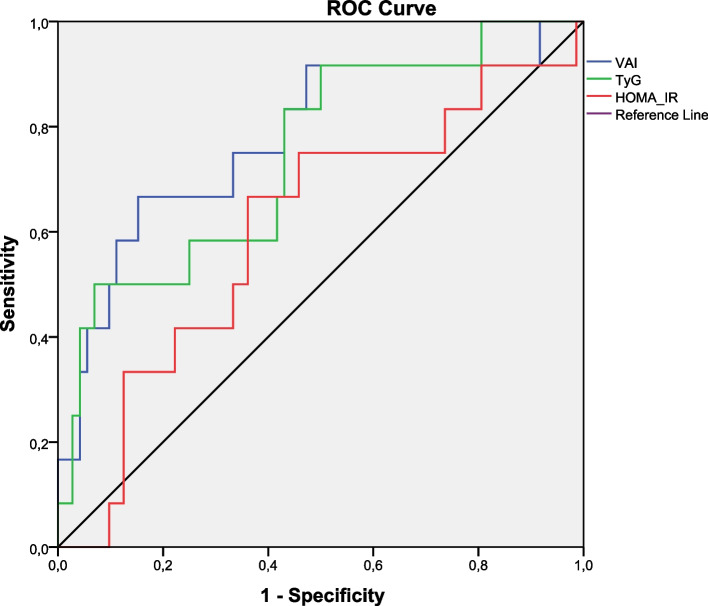
Receiver-operating characteristic curves to predict MetS by HOMA-IR, TyG, and VAI in boys with obesity

**Fig. 2:**
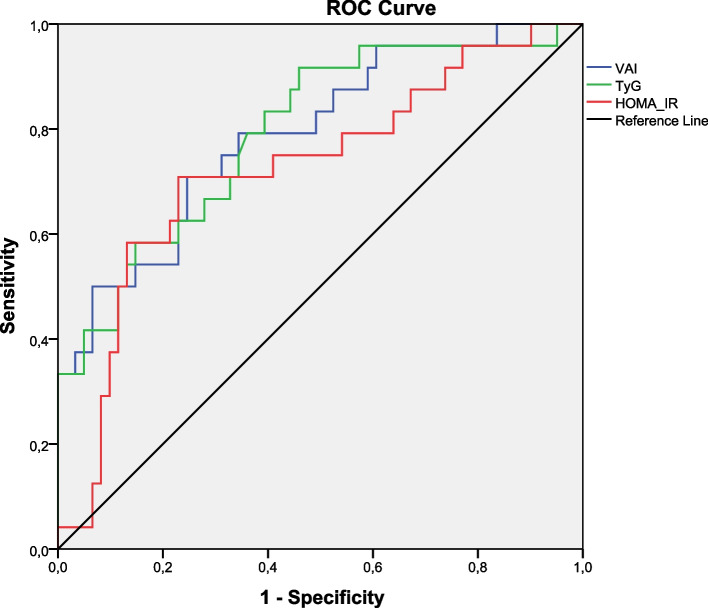
Receiver-operating characteristic curves to predict MetS by HOMA-IR, TyG, and VAI in girls with obesity

The abilities of the triglyceride-glucose index, visceral adiposity index, and HOMA-IR index for predicting the MetS were evaluated by ROC curve analysis for boys and girls, and the results were presented in Figs. 1 and 2, respectively.

The cut-off points of the TyG, the VAI, and the HOMA-IR index with the best balance between sensitivity and specificity for MetS and the area under the curve (AUC) were presented in Table 3. Cut-off values of all three indices were found to be higher in girls than in boys. The AUC values of both TyG and VAI index cut-off points were significantly higher than the AUC value of the HOMA-IR cut-off value in both sexes (*p* < 0.01).

**Table 3:** The cut-off points of the TyG, VAI, and HOMA-IR Index by gender for predicting MetS

**Gender**	**Indexes**	**Sensitivity–Specificity**	**Cut-off point**	**AUC**^a^	**95% CI**^b^	***p***^‡^
Boys	HOMA-IR	0.67–0.64	4.127	0.605	0.429–0.781	0.245
	TyG	0.74–0.57	8.322	0.747	0.594–0.899	0.006
	VAI	0.75–0.68	2.724	0.779	0.622–0.935	0.002
Girls	HOMA-IR	0.71–0.70	4.775	0.725	0.600–0.850	0.001
	TyG	0.74–0.66	8.507	0.793	0.685–0.901	0.000
	VAI	0.75–0.69	4.289	0.790	0.682–0.899	0.000

^a^Area Under the Curve

^b^Confidence interval

^‡^probability; *HOMA-IR* Homeostasis Model Assessment Insulin Resistance, *TyG* Triglyceride-glucose index, *VAI* Visceral adiposity index

## Discussion

Cardiometabolic disorders are important public health problems due to their negative effects on disease burden and healthcare costs. For this reason, many studies have been conducted on cardiometabolic risk factors and early detection criteria in children and adolescents as well as adults [4]. VAI and TyG index are valuable tools to identify MetS and estimate cardiometabolic risk factors in children and adolescents with obesity because both have higher values in children with obesity, and are advantageous as an easy tool for detecting MetS [20, 26]. In addition, many researchers have declared that the association of VAI with metabolic diseases in adults is stronger than with traditional anthropometric indicators of adiposity, and its strength in children and adolescents continues to be investigated [12, 15, 26].

Studies conducted in different countries reported that the cut-off points ​​calculated for VAI in predicting the MetS in children ranged from 1.30–6.15 (AUC: 85%-77%) in boys, and 1.78–4.95 (AUC: 89%-76%) in girls [12, 15, 16, 26]. Our study was conducted on Turkish children with obesity in a more limited age group, and the cut-off points ​​we obtained for VAI were found higher than in the aforementioned studies. The presence of a hypersensitive hypothalamic–pituitary–adrenal axis in children with obesity in the prepubertal-pubertal period negatively affects the hormonal balance. The balance between the lipogenic effect of cortisol and insulin, which are hormones effective on body fat distribution, and the lipolytic effect of sex steroids and growth hormones can be disrupted, especially in children with obesity [27]. The differences between sexes are related to the differences in the amount and location of visceral fat accumulation, as well as hormones [28]. The reason for the high VAI cut-off points and the lower sensitivity/specificity values determined in our study ​​may be due to the selection of the sample from the age group close to the pubertal period, as well as the differences in the distribution of adipose tissue in children with obesity. In a systematic review, it has been reported that the distribution of visceral adipose tissue in children and adolescents is associated with factors such as genetics, ethnicity, gender, age, developmental level, and puberty [29]. Therefore, these factors should be considered when evaluating predictors of cardiometabolic risk. For the measurement of waist circumference, which is also a predictor for the MetS, it is recommended to use percentiles specific to ethnic origin, in addition to age and gender [30]. The main factor that makes VAI an important predictor is that it is an indicator of early cardiometabolic risk when the metabolic syndrome does not show clinical signs yet. Having better performance of VAI in predicting the MetS has been explained by the association of VAI with three main components of the MetS criteria, WC, TG, and HDL, and adiponectin and insulin resistance, which are cytokines associated with high levels of inflammation [27].

However, there are some studies in the literature, suggesting that the TyG index is a better indicator than the VAI in adults [31–33]. The TyG index is associated with insulin resistance and dyslipidemia and has been recommended as a good indicator of the presence of cardiometabolic risk factors, particularly insulin sensitivity [11, 18, 34, 35]. Besides, it has been reported that the TyG index has a high sensitivity for MetS in children and adolescents with obesity [11, 19, 36]. Similar to our result, Brito et al. [20] reported in a systematic review that in the ROC curve analyses performed to predict the cardiometabolic risk factors, the TyG index threshold values ranged from 4.65 to 8.66, and the cut-off point in girls was higher than in boys. Similarly, it was stated that the cut-off points ranged between 8.52–8.74 (AUC: 84%- 90%) while the sensitivity (82%-92%) and specificity (82%-91%) values were high in another study also [36]. Although we obtained lower sensitivity and specificity values compared to these studies, their cut-off points are consistent with our cut-off points for both genders. It has been stated that the difference between the cut-off points may be originated from age, gender, ethnicity, pubertal level, diet, and the TyG index formula used. [7, 18, 20, 35, 37]. We consider that the main reason for reporting different cut-off points for TyG is the difference in the TyG index formula used. Because, when the TyG index is calculated according to the original formula (Ln [fasting triglycerides (mg/dL) x fasting glucose (mg/dL) /2]) results in close to eight, while the following formula gives a result in close to four: Ln [fasting triglycerides (mg/dL) x fasting glucose (mg/dL)]/2. Unfortunately, the studies that have correctly computed and reported the TyG index using the original formula are limited.

Although there is an increasing number of studies detecting IR and related metabolic abnormalities in children, the HOMA-IR threshold value has not been determined for metabolic syndrome, but a positive correlation of HOMA-IR scores with BMI has been demonstrated [25]. In a systematic review, it was reported that the cut-off points of HOMA-IR in children and adolescents for predicting the MetS ranged from 2.30 to 3.59, and the sensitivity and specificity of these values were 59%-89% and 62%-87%, respectively [10]. In a study conducted on obese Turkish adolescents, the cut-off point for HOMA-IR was determined 2.62 in boys (AUC = 0.710; sensitivity = 80·4%; specificity = 47·4%), and 2.66 in girls (AUC = 0.673; sensitivity = 81.8%; specificity = 40.6%) [11]. The cut-off points of HOMA-IR were approximately twice as high in our study, but lower sensitivity and higher specificity were determined. These different findings indicate the existence of other factors other than ethnicity or nutritional culture. For example, the mean age of our study group is lower than those of many other study groups. It could not be shown clearly there is a correlation between visceral adiposity and inflammation markers, and insulin sensitivity in the pediatric age group, and the genetic factors have a greater role in emerging metabolic risk [38]. However, it was also noteworthy that a significant cut-off point for HOMA-IR has been found only in girls. Previous research has shown that adipose tissue produces adipokines that play a key role in the development and progression of insulin resistance and plays an important role in energy regulation through endocrine, paracrine and autocrine signals [39]. Therefore, it is hypothesized that adipokines have a possible link between obesity and other risk components of metabolic syndrome. An increase in adiponectin plasma concentration decreases insulin resistance, while leptin leads to the development of insulin resistance [40]. In studies conducted on people with obesity, serum leptin / adiponectin levels were found to be significantly higher in women than in men [41, 42]. Ather et al. [43] drew attention to a positive correlation between leptin and overweight in adolescent girls, and a negative correlation with adiponectin, besides to higher leptin levels in children with metabolic syndrome. In addition, it was shown that age and sex were significantly associated with MetS, and MetS prevalence generally increased with age in children [8]. All these findings explain why HOMA-IR levels and cut-off values for MetS are different in boys and girls with obesity.

Another disadvantage of HOMA-IR is its limited usage due to cost, requiring standardization for insulin, a labile hormone [19, 34]. It was shown that the difference in insulin sensitivity in children with obesity between the sexes is due to phenotypic characteristics, that there may be a gene responsible for it, and that girls have higher insulin resistance [44]. A study conducted in the Turkish population found that the FTO rs1421085 variant was associated with the risk of obesity only in women [45]. All these findings show that longitudinal and genetic studies are still needed to ensure standardization of HOMA-IR's cut-off points according to age, sex, ethnicity, and obesity status and to confirm its clinical benefit.

In contrast, the VAI and the TyG index both had significant cut-off values in both sexes, and their AUC values were larger than that of HOMA-IR. The TyG index has been proposed as a good indicator of insulin sensitivity, as it has been found significantly associated with glucose metabolism rates in many populations [11, 18, 34, 35]. In addition, some studies have suggested that TyG is a better indicator than VAI also in adults. [32, 46]. Nevertheless, it should be taken into account that in studies that indicate that VAI or Tyg is a superior predictor, the male/female ratio of participants may have affected the results.

There were some limitations to our study. First, although euglycemic hyperinsulinemic clamp technique is the gold standard method, we used the HOMA-IR to determine insulin resistance, as the former is a time-consuming, expensive, and invasive method. Second, the participants were limited to children aged 9–10 years in Samsun province, therefore the results may not be generalizable to other populations. Besides the cross-sectional study design, having not assessed the children's diet and physical activity were also other limitations.

## Conclusion

Based on our results, the VAI had the highest area under the curve in boys, while TyG had the highest AUC in girls, suggesting both are more useful predictors than the HOMA-IR to determine for MetS risk. We determined that the VAI and the TyG index are effective indicators in the light of significant results obtained by ROC analysis in assessing the MetS risk in children with obesity. Both indexes can be considered useful tools in pediatric research and the evaluation of interventions. However, the HOMA-IR index formula needs to be developed taking into account age, gender, and ethnicity. For this purpose, it is thought that there is a need for cohort studies to be conducted in different populations and age groups.

## Data Availability

The datasets used and analyzed during the current study are available from the corresponding author on reasonable request.

## Abbreviations

BP: Blood pressure
CVD: Cardiovascular diseases
DBP: Diastolic blood pressure
FBG: Fasting blood glucose
HDL: High-density lipoprotein
HOMA-IR: Homeostasis Model Assessment Insulin Resistance
IFG: Impaired fasting glucose
LDL: Low-density lipoprotein
MetS: Metabolic syndrome
SBP: Systolic blood pressure
TC: Total cholesterol
TG: Triglyceride
TyG: Triglyceride-glucose index
VAI: Visceral adiposity index
WC: Waist circumference
